# Social Policy Responses to the Covid-19 Crisis in China in 2020

**DOI:** 10.3390/ijerph17165896

**Published:** 2020-08-14

**Authors:** Quan Lu, Zehao Cai, Bin Chen, Tao Liu

**Affiliations:** 1 China Social Security Research Center, Renmin University of China, Beijing 100872, China; hanluquan@126.com; 2 School of Public Administration, China University of Labor Relations, Beijing 100045, China; 3 School of Sociology, Huazhong University of Science and Technology, Wuhan 430074, China; chenbin2020@hust.edu.cn; 4 School of Public Affairs, Zhejiang University, Hangzhou 310058, China; 5 Center of Social Welfare and Governance, Zhejiang University, Hangzhou 310058, China; 6 Institute of East Asian Studies, University Duisburg-Essen, 47057 Duisburg, Germany

**Keywords:** Covid-19, social policy, responses, intervention, state

## Abstract

The 2020 coronavirus pandemic has catapulted China into a serious social and political crisis. This article focuses upon how Chinese social policy has responded to the Covid-19 crisis. It reveals that the Chinese welfare state has woven a comprehensive social safety net to mitigate the social suffering of Chinese society in the mid- and post-crisis periods. Different types of social policy programs have been combined and synthesized, including social insurance, social assistance, and social welfare arrangements. Facing the challenges of the new risks caused by the pandemic, the collaboration of the Chinese state and intermediary social welfare organizations has played a crucial role in providing both cash benefits and social services (benefits in kind). For the first time, social policy in China has acted as a major player for coping with the negative outcomes of a pandemic. This article concludes that the pandemic-related crisis has justified an interventionist approach and logic, driven by the state’s welfare system, which favors a model of “big government”. However, this model also requires justification and legitimation.

## 1. Introduction

The Covid-19 pandemic represents the most serious public health crisis in China since the founding of the People’s Republic of China (PRC), with the fastest spread and the widest infection range, challenging the country’s socioeconomic development and people’s daily life. Facing these challenges, Chinese governments have established strong command-and-control mechanisms, reminiscent of war times, to respond to this crisis and control the virus [1]. These measures appear to have worked sufficiently, and the pandemic in China seems to have been brought under control. The central government has played a proactive role by issuing a number of key policies in the field of social security, effectively relieving the anxiety of patients infected with Covid-19 and their families from the financial burden of medical treatment, and also fully mobilizing social resources to effectively support the resumption of work and production.

**Figure 1:**
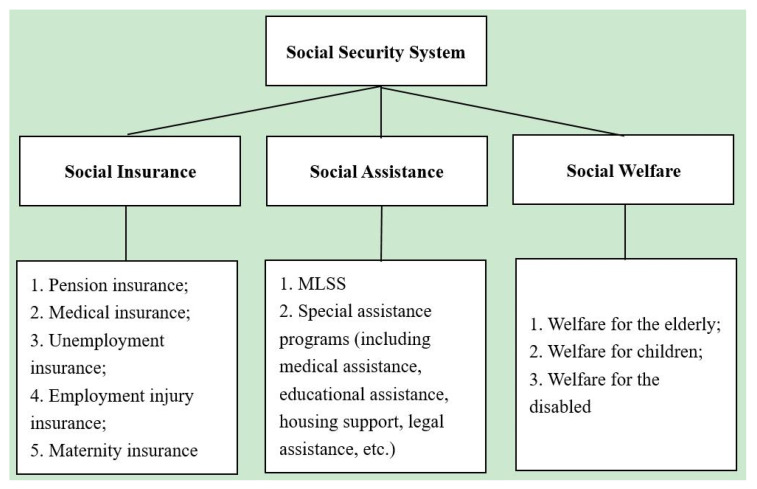
The social security system in China. Source: Authors’ own compilation.

China has witnessed a rapid expansion of social security programs over the past decades, especially since 2000, and has established the world’s largest comprehensive social protection network [2]. Currently, the social security system in China consists mainly of three types of social programs: contributory social insurance, non-contributory social assistance, and tax-financed social welfare. The first includes pension insurance, medical insurance, unemployment insurance, employment injury insurance, and maternity insurance, and all of these programs are extended to urban employees. At the same time, it also includes basic pension insurance and basic medical insurance for urban and rural residents who do not fall under the category of urban employees. According to a report released by the Ministry of Human Resources and Social Security, the total number of persons covered by pension insurance is 943 million (419 million are urban employees and 524 million are urban and rural residents). The total number covered by medical insurance is 1.344 billion (317 million are urban employees and 897 million are urban and rural residents). More than 95 percent of Chinese citizens are covered through this program. Medical expenses in conformity with the drug catalogue, diagnosis and treatment items and medical care service facility standards for medical insurance and medical expenses for emergency treatment or rescue are paid from this program. The current reimbursement rate is around 60 percent of the total expenses for insured urban employees and about 50 percent for insured urban and rural residents. Further, at the end of 2018, 196 million employees had been covered by unemployment insurance, 239 million employees had been covered by work accident insurance and 204 million employees had been covered by maternity insurance. For China’s social assistance system, the most important program is the Minimum Living Standard Scheme (MLSS, known as *Dibao*), which covers residents whose per capita income/annual net income falls under the threshold of the local minimum living standard in both urban and rural areas. According to the Statistical Report on the Development of Civil Affairs issued by the Ministry of Civil Affairs in 2019, 10.07 and 35.02 million people received benefits from the MLSS in urban and rural areas, respectively. In addition, medical assistance, educational assistance, housing support, legal assistance, and a relief system after natural disasters are also important ingredients of China’s social assistance system. The country’s social welfare program provides funds and social care services to ensure the livelihood of the elderly, children, and persons with disabilities who experience extraordinary difficulties [3] (see Figure 1).

However, interventions through social protection policy and questions that must be reconsidered in relation to disease-related crises have been largely neglected in academic research. Therefore, this article will specifically analyze how different types of current social security programs have responded to the outbreak and the outcome of the pandemic-related crisis and highlight the shortcomings of these measures. The remainder of this article is organized as follows. Section 2 constructs an analytical framework for the study. Section 3 presents the research findings, including reflections on policies in areas such as unemployment insurance, welfare institutions, social insurance, and social assistance. Section 4 offers a discussion and concludes the paper.

## 2. Analytical Framework: Crisis-Related Social Policy and Social Protection

Within modern capitalist welfare systems, social policy is considered an institutional response to the negative effects of free-market competition and the rising and contingent risks that occur during market fluctuation [4,5,6]. In particular, state-organized social protection programs have corrected the primary distribution mechanism, mitigating social problems in the capitalist market economy. Social policy dovetails with the long-term institutional policy intervention by welfare states in the field of production and reproduction [7], having been established in the continuing transformation of the capitalist economy from a laissez-faire model to a market economy increasingly regulated by welfare states [8]. Although the formation of modern state-organized social policy represents a long-range transitionary process, it is necessary to take another aspect of social policy into consideration—the social policy and social protection adopted and stimulated by short-term events and shocks. In particular, special attention should be paid to social crises and socially and economically anomic developments that deviate from the state of social normality, challenging and jeopardizing regular and consistent socioeconomic development within a short-term or medium-term period [9,10]. Social crises often alter the “normal” state of a society, creating a critical juncture posing an acute threat to the status quo of the current social and political system. The nexus between crises and social policy has not been investigated sufficiently in academia. Commonly, crises have been regarded as negative events that can overshadow the regular functioning of welfare states, causing dysfunctionality and disruption of the existing social order. Moreover, historically, social crises are closely related to welfare state retrenchment. For instance, the oil crisis in the 1970s and the subsequent stagflation in the Western world had precipitated the end of the golden age of capitalism and the postwar economic boom, resulting in the reduction of social expenditures during the Reagan and Thatcher administrations [11,12].

However, the intuitive assumptions concerning the nexus between crisis and welfare retrenchment have been upended by empirical developments in various welfare states and welfare regimes. Usually, social crises have increased the suffering and the poverty gap in a society and precipitated social protests and social turmoil, challenging the current social political order and endangering the legitimacy of governments. Against this backdrop, many state administrations have been inclined to expand public revenues and state social investments to circumvent social disorder and sharply increasing social and economic problems. In some cases, crises have fostered new opportunities for the extension of welfare state intervention and the expansion of social protection programs. Conflict theories have generally verified that social conflicts such as social protests, social movements, and struggles among different social classes for resources have benefited and accelerated social policy expansion in welfare states [6,13]. Social crises represent a special type of social conflict. During the outbreak of crises, social conflicts usually intensify, social grievances are raised, and the battle among different social classes for subsistence and resources escalates. All this tremendously jeopardizes social cohesion; thus, social and political responses are urgent for the survival of governments. If governments do not act in time, social discontent may transform into destructive protest. For instance, the Roosevelt-administration initiated welfare state arrangements in the United States in the 1930s after the shocking (after-) effects of the Great Depression began to unfold [14]. Hort and Kuhnle have verified empirically that after the Asian financial crisis of 1997–1998, social expenditures in the region of East and Southeast Asia were not reduced as usually assumed; on the contrary, in nearly all of the 10 selected examples in this region, social protection programs remarkably expanded, during and even after the crisis [15]. These counterexamples demonstrate that crises do not always cause austerity and the state’s retreat from public investment. Under certain conditions, crises and the subsequent pressure imposed upon governments may unintentionally become driving forces for elite groups to change and respond to critical demands from society. In other words, the suffering during periods of crises can be transformed into positive assets for welfare state extension and expansion. This paradoxical development concerning welfare expansion during economic crises has also been traced in the developmental trajectory of the Korean welfare state [16].

Historical examples, along with the current pandemic crisis in 2020, have unveiled a trend in which state administrations from various countries are inclined to expand their capacities and intensify state intervention in society to lessen social tensions and calm the social discontent and public anger emerging from the outbreak of crises. In major global crises, such as the Great Depression, the global financial crisis of 2007–2008, as well as the Covid-19 crisis, “big government” represents a model for coping with social problems and social conflicts [17]; accordingly, the myths of market omnipotence and neoliberal ideology have been substantially diluted [18]. Counter to assumptions concerning the retreat of welfare states, these substantial crises have strengthened the power of the state, disenchanting the “laissez-faire model”. Facing a crisis of acute existential survival, ordinary working populations and residents, as well as enterprises short on liquidity must seek assistance and bailouts from the state. In turn, the state must devote considerable resources to society to ensure a threshold of existence. Otherwise, widespread malnutrition or famine, or the demise of many small- and medium-sized enterprises, would very likely endanger the state’s basic functioning, causing enormous loss of human resources and financial drain, and leading to danger and risk for the survival of the state itself. Crises have provided stimuli for governments to assume the role of the ultimate guarantor and provider of public goods for their citizens. Thus, crises are somehow related to “big government” and an active interventionist state. In the Chinese case, the pervasive model of “big government” may shift rapidly into a quasi-war state to respond to crisis. However, many emergency measures and acts need special justification and legitimation, since personal freedoms and right of free mobility may be constrained, as they have been during the pandemic-related lockdowns and curfews.

The social security system has been designed and built to cope with risks. However, the Covid-19 pandemic is a sudden public health event, which by itself is a risk of a new type. It therefore brings substantial challenges to the existing social security system. During the Covid-19 pandemic, China’s social security system has largely functioned well in many aspects, but some serious problems have also been exposed that call for further improvement.

This study applies the method of event analysis with a special focus on social and political responses to this special crisis event. The trajectory and chronological development of Covid-19 from February through June of 2020 has been intensively observed and consequently integrated into our analysis of the social policy responsiveness to this crisis-related event. The pandemic-related crisis has been divided into three primary stages—the pre-crisis, during crisis, and post-crisis periods. Regular social policy arrangements before the outbreak of Covid-19 have previously been introduced. Some hallmark subevents in the arena of Chinese social protection during the outbreak of the pandemic and in the post-crisis period have been identified and analytically discussed and reflected, including policy measures and strategies adopted in the areas of health insurance, unemployment insurance, social assistance, and social welfare, among others. To complement our event-centered policy analysis, we have collected different kinds of secondary data such as policy documents and data published by the state administration, including the Ministry of Human Resources and Social Security (MHRSS) and the Ministry of Civil Affairs (MCA), which characterize the coping strategies of the Chinese state and society in relation to Covid-19. Secondary documents issued by different state administration and public media have uncovered the urgency and exigency created by pandemic and presented an assemblage of emergency measures, formal and institutional policies implemented during the crisis. Through the process tracing crisis-related events, using various data sources, we reconstruct the panorama of social policy responses to the critical juncture that Chinese society experienced amid the pandemic.

## 3. Findings

### 3.1. Unemployment Insurance: Functions Must Be Improved and Beneficiaries Must Be Expanded

The impact of the Covid-19 pandemic on employment might be short-term, but it is more serious and complicated than the situation of SARS in 2003 [19]. Therefore, helping enterprises to overcome difficulties is still an important measure to stabilize employment and the economy. Governmental departments have formulated various policies for different regions and different types of enterprises to reduce social insurance contributions. For example, in Hubei Province, where the pandemic has been most severe, the government has exempted certain employers (except for the public sectors) from paying social insurance fees for no more than five months. For other regions, the social insurance contributions of large enterprises may be halved for a period of no more than 3 months, not exceeding 5 months for small- and medium-sized enterprises. Finally, enterprises with difficulties in production and operation may apply for deferred payment of social insurance premiums for no more than 6 months. (The above contributions refer to the portion paid by the employer; payments by individuals must be made on time. It is noteworthy that none of the above policy adjustments affect personal entitlements.)

Using unemployment insurance to help employers and reduce the unemployment rate is another important measure. Specifically, for small- and medium-sized enterprises, if the unemployment rate is not higher than that in the national survey of the previous year, part of the unemployment insurance benefits may be refunded to stabilize employment. Meanwhile, enterprises were encouraged to implement training programs for those affected by the pandemic, which could be subsidized if they organized employees to participate in offline or online vocational training during the shutdown period.

According to a spokesperson from the Ministry of Human Resources and Social Security, the pension insurance, unemployment insurance, and employment injury insurance contributions reduced in February reached 123.9 billion yuan ($17.5 billion). The total amount of deductions in contributions from February to June was estimated to be more than 500 billion yuan ($70.6 billion), effectively supporting the resumption of work and production. At the same time, 1.46 million enterprises received unemployment insurance refunds, amounting to 22.2 billion yuan ($3.1 billion), benefiting 49.51 million employees [20].

Notwithstanding the achievements outlined above, some problems have also been exposed with regard to supporting enterprises by fully utilizing unemployment insurance policy. The functions of the unemployment insurance system are twofold: one is to maintain the basic livelihood of the unemployed and their families, and the other is to actively create conditions for their reemployment through professional training, job referrals, and other means. At present, the main function of this system in China is to maintain the livelihood of the unemployed; however, the promoting and preventing functions have not been fulfilled. It is mandated that employees shall participate in unemployment insurance, and the premiums should be jointly paid by employers and employees; however, statistics reveal that the number of people participating in this insurance program stands at 196 million, accounting for only 45 percent of urban workers. This is the lowest insured rate among all types of social insurance, because a large number of migrant workers and informal employees are not included in this program. At the same time, the cumulative balance of unemployment insurance funds reached 581.7 billion yuan ($82.1 billion), 6.4 times of its expenditure of 91.5 billion yuan ($12.9 billion) in 2018 [21]. In other words, even if unemployment insurance fees are no longer collected, the funds can still be used for more than 6 years, suggesting that the amount of cash benefits paid out is low, and other unemployment benefit programs should be added. It is worth noting that, in this pandemic, some companies in China have adopted innovative forms of employment such as “shared employees”, with individuals working in multiple companies at the same time, to reduce their own expenditure burden by lowering wages and/or not paying social security fees; however, the government has also required these companies to reduce the layoff rate and pay basic wages. Therefore, a responsibility-sharing mechanism should be established to finance the expenditures. In this sense, the unemployment insurance funds can shoulder the task by paying a portion of the wages for workers who have not returned to work, and thereby reducing the burden on the enterprise. These measures will better allow the enterprises to weather the storm.

The practice of the unemployment insurance system has exposed another problem: according to current regulations, insured persons will not be qualified to receive unemployment insurance benefits after being laid off, unless they have contributed to the system for no less than one year. In the pandemic, a new special policy has been introduced to provide unemployment subsidies for laid-off employees who have contributed for less than one year. However, this subsidy is lower than the usual unemployment insurance benefits. We believe that the special policy should become part of the formal unemployment insurance system and be incorporated into the current regulations.

### 3.2. Welfare Organizations: Closed Management Has Mixed Advantages and Disadvantages, and a Social Compensation System Must Be Established Urgently

Welfare organizations, such as nursing houses for people in need, tend to be higher-risk areas in terms of emergency management, as these are places where elderly people, dependent children, people with disabilities, etc., live in close quarters. In the event of emergencies (including the Covid-19 pandemic), high-density living spaces and collective actions cause a chain reaction of infection, making residents even more vulnerable [22]. Indeed, specific demographic groups living in welfare organizations are susceptible to Covid-19 because of their underlying health conditions, making special protection policies during the pandemic more significant and urgent than ever.

In the early stages of the areas with high incidence of Covid-19 in China, there were clustered cases within nursing homes. On 21 February 2020, according to the notice of the Wuhan Civil Affairs Bureau, as of February 19, social welfare agencies in Wuhan had a total of 12 confirmed cases; 11 were elderly residents (including one deceased) and one employee [23]. In response to this, the relevant departments implemented three measures: (1) Several anti-pandemic guidelines were issued for different types of welfare agencies based on the risk level of the region, followed by stricter closed management for these agencies to accommodate the elderly, persons with disabilities, and their service staff. (2) Cross-regional caregivers were arranged to provide necessary support. There were more than 20,000 elderly residents in the old-age care agencies in Wuhan, but only 3000 local care staff. In addition, some of these caregivers were infected or self-isolated, so there was a shortage of care staff in general. Thus, the Ministry of Civil Affairs of the central government coordinated cross-regional care and nursing staff to offer support to Wuhan [24]. (3) Care services for stay-at-home elderly residents whose families were isolated and/or sick were provided. Daily life became more complicated for elderly residents living alone due to the pandemic, and their routine caregivers were not always available to serve in a timely manner. Moreover, some caregivers were being treated or medically isolated because of the pandemic, which forced vulnerable groups to stay at home. To address this situation, welfare agencies would provide door-to-door services or arrange for these residents to be cared for in-house.

However, the protection for some caregivers and volunteers in this case was, to some extent, less focused. In the process of prevention and control of the Covid-19 pandemic, in addition to medical workers, some volunteers were infected or even passed away at work, but technically, volunteers cannot be identified as beneficiaries of work-related accident benefits; similar cases also applied to caregivers as informal workers without work-related accident insurance. Therefore, establishing a social compensation system is necessary. Social compensation refers to the compensation by the state and society for the loss of interests (physical disability or a sharp decrease in income, and so on) of relevant stakeholders, when is caused by uncontrollable risks (such as natural, societal, or policy-related events). Germany and the Taiwan region have built relatively complete social compensation legal systems, but mainland China has not yet established such a system. Unlike the civil compensation caused by torts, the administrative compensation brought by wrong administrative acts and state compensation brought by judicial misconduct, social compensation is mainly to pay for losses caused by wars, natural disasters, and other uncontrollable social risks. This includes not only compensation for the direct victims of such disasters, but also praise for the staff and volunteers involved in disaster relief.

### 3.3. Social Insurance: The System Designed to Deal with Risks Faces New Risks and Challenges

In China, the social insurance system is primarily composed of five sub-programs: medical insurance, employment injury insurance, unemployment insurance, pension insurance, and maternity insurance. The first three in particular have played a major role in providing economic support for the insured during the Covid-19 pandemic.

#### 3.3.1. Medical Insurance and Related Policies: Programs That Bear the Brunt

As mentioned above, there are two kinds of medical insurance system in China: one for urban employees, and the other for urban and rural residents other than urban workers. The former is paid by employers and employees (6 percent and 2 percent of wages, respectively), and the latter is paid by residents and subsidized by the government (in 2020, the individual contributions are 280 yuan, and the central and local financial subsidies are not less than 270 yuan). The medical insurance fund is principally used for in-hospital and substantial medical expenses of the insured, and the current average reimbursement rate is more than 70 percent of the total expense for urban employees, and around 60 percent for urban and rural residents who participate in the scheme.

Effective medical security measures, mainly medical social insurance, were taken in a timely manner during the Covid-19 crisis in China, so that patients and their families are relieved from worrying about treatment costs, specifically: (1) Shortly after the outbreak, the state issued a policy to include drugs and medical services for the treatment of the new coronavirus as part of the payment range for the medical insurance fund. (2) Furthermore, the personal medical burden is borne by fiscal resources of the government, thus achieving free treatment for patients with Covid-19. The policy was initially limited to confirmed cases, but was later expanded to suspected ones. (3) Simultaneously, when people seek medical treatment within one’s coordinated areas of the social medical insurance fund, it is mandated that treatment must be provided first, with the fee to be settled later. (4) For medical institutions admitting a large number of patients, social medical insurance would prepay funds to ensure that the effectiveness of treatment from hospitals is not impaired due to payment policies.

The above policies may be summarized as “two guarantees”, first to ensure that no patient is rejected or treated in an untimely manner due to medical expenditure or cost problems, and, second, to guarantee that no designated medical institution is impaired from treating patients due to budget management regulations from the medical insurance fund. For foreign patients, if they have participated in China’s basic medical insurance or any commercial insurance, their fee will be paid by the corresponding insurance funds; alternatively, they must bear the cost themselves. However, whether insured or not, medical institutions will treat first and charge later to ensure that everyone can receive medical treatment in time.

The total cost of confirmed and suspected Covid-19 cases in China was about 1.486 billion yuan ($0.21 billion) as of 6 April 2020. The per-capita medical cost of diagnosed inpatients reached 21,500 yuan ($3035), and of the severe ones, more than 150,000 yuan ($21,176). The medical insurance fund paid 990 million yuan ($139.8 million), accounting for 66.6 percent of the total medical cost. The total cost of diagnosed inpatients involved was 1.118 billion yuan ($0.16 billion), 746 million yuan ($105.3 million) of which was paid by the medical insurance fund, accounting for 66.7 percent. The total cost of the suspected patients was 368 million yuan ($52 million), with the medical insurance fund paying 66.6 percent of it (245 million yuan) [25]. Overall, the medical insurance fund paid about two thirds of the total cost, and the remaining one third was borne by fiscal funds at various levels.

However, these medical security measures were not without constraints. Many of the medical security policies issued during the Covid-19 pandemic were interim measures, which made them difficult for some local medical funds to implement in an orderly fashion [26]. One question yet to be clarified concerns the ultimate responsibility for payment: between public finance and the medical security fund, which one should eventually bear the medical expenses, or how they should be shared on the basis of specific principles? That is, in this pandemic, the medical security funds and fiscal funds work together to provide free medical care to patients. However, according to the Social Insurance Law (the third paragraph of Article 30), the basic medical insurance funds should not pay expenses that should be borne by public health funds. Therefore, it is necessary to rethink the relationship between public finance and medical security funds in major pandemics like Covid-19.

To address this problem, the first step is to take the perspective of the comprehensive process of public health emergency management. According to China’s Emergency Response Law, the emergency management process includes four phases: prevention and preparation; monitoring and early warning; rescue and disposal; and rehabilitation and recovery.

On the one hand, the beneficiaries of the public health funds include the entire population, so these funds should be mainly used in the prevention and preparation phase, for vaccination, tracking, and service provision for people who test with underlying health conditions. On the other hand, medical insurance helps diversify risks among insured persons, so it should mainly target the medical expenses of patients.

Furthermore, the impact of major public health events on different regions is often disproportional. According to the latest information, among 84,614 confirmed cases recorded nationwide, 68,135 were diagnosed in Hubei Province, accounting for more than 80 percent of the total cases. However, at present, the overall planning level of medical insurance funds in China is only at the municipal level, inevitably resulting in a larger burden of medical expenses in areas with severe pandemics, which cannot be shared on a larger scale. Faced with such a contradiction, limited by the reality of the situation, medical insurance cannot achieve national pooling in a short period of time; it is still necessary to establish a sharing mechanism between the medical insurance and the public finance for medical expenses, especially in areas with severe pandemics.

The responsibility-sharing mechanism between public finance and the medical insurance system also concerns the issue of due beneficiaries, or more specifically, foreigners in China and Chinese students overseas. For foreigners in China, as long as they have participated in medical insurance, they are entitled to benefits; and if they also fulfill their tax obligations, they should also benefit from public financial subsidies. For overseas students, in the context of the global spread of the Covid-19 pandemic, a large number of students want to return to China. In response, the Chinese government has issued a policy stating that if they return, they will be responsible for their accommodation and boarding expenses during the period of quarantine. Whether their medical expenses will be paid depends on whether they have participated in medical insurance. This practice is in line with the basic principles of medical insurance. However, if the medical expenses are mainly to be borne by public finance at the national level, these students should enjoy free treatment, regardless of whether they are insured or not, paying taxes or not.

#### 3.3.2. Employment Injury Insurance and Unemployment Insurance: Social Protection Policies That Played an Active Role

Other insurance sub-programs have also contributed to the battle against the Covid-19 crisis. As far as unemployment insurance and work-related injury insurance are concerned, although the contribution rates have continued to decline over the past five years, and the scope and level of expenditures have increased, the accumulated balances have continued to rise [27]. (There may be many factors contributing to the increasing accumulated balance. First, and likely foremost, more workers have participated in the social insurance system. According to the Ministry of Human Resources and Social Security, the number of participants in the unemployment insurance system increased from 170.43 million in 2014 to 196.43 million in 2018. Second, on the expenditure side, the total number of beneficiaries has remained stable, fluctuating around just above 2 million people during 2014–2018, despite the relatively higher level of benefits and larger scope of compensation projects for qualified insured persons. Moreover, the level of overall planning in the unemployment insurance system has been low, reducing the efficiency of unemployment insurance funding, which also helps explain the situation. Relatively developed regions usually enjoy more advantages in the labor market such as more local and immigrant workers, and thus more contributions to the unemployment insurance program, while unemployment rates are relatively low, in contrast to underdeveloped regions. These developed regions have contributed the most to the accumulated balance on a national scale, but this surplus cannot be properly shared by other regions if they are not in the same overall planning area.) This has provided a solid foundation for expanding expenditures related to the pandemic. Employment injury insurance was adjusted in time to recognize and thus protect caregivers on the front line. To begin with, it was made clear that medical care staff and other related staff, who were infected with Covid-19 or died from it in the course of their work to prevent and control the pandemic, would be recognized as work-related injuries cases, and their legitimate entitlements and interests would be protected. Given that the Covid-19 outbreak in China was mainly concentrated in Wuhan, the central government mobilized medical care staff from other administrative areas to support Wuhan City. From 24 January (a traditional Chinese holiday, the eve of the Spring Festival) to 8 March, approximately 42,600 staff members from 346 medical teams across the country arrived in Wuhan and Hubei Province to participate in medical treatment. However, as of 11 February, 1716 medical care staff, accounting for 3.8 percent of the national confirmed cases, had been confirmed as infected with Covid-19 nationwide [28]. In addition, as of 8 March, 53 community workers perished in the line of duty during the epidemic prevention and control [24]. In order to ensure the basic entitlements for these employees, the relevant authorities opened a “green channel” to simplify the procedure for identifying work-related injuries. At the same time, if the requirements are met, deceased medical care staff and other pandemic prevention workers are eligible to be recognized as martyrs. According to the Regulation on Honoring Martyrs, the state has established a reward system for martyrs. The reward standard is 30 times the per capita disposable income of urban residents in the previous year. Meanwhile, the state grants preferential treatment to the survivors of martyrs to ensure that their quality of life is not lower than the average living standards of local residents, and the state will also provide support for their children’s education and parents’ pension.

Furthermore, the unemployment insurance system, by nature, works counter-cyclically in the sense of economics. As the pandemic caused a large number of enterprises to fail in resuming a timely production schedule, workers were thus unable to return to work, which adversely affected both the employers and employees. As a policy response, a series of regulations have been issued by various governmental departments to broaden the scope of beneficiaries. For insured persons who lost their jobs due to the pandemic in Hubei and other pandemic-stricken areas, if they do not meet the basic requirements for receiving unemployment insurance (e.g., participating in and contributing to the insurance fund for no less than one year), they will be offered an unemployment subsidy. The standard is no higher than 80 percent of the local unemployment insurance premium, and it is only paid for six months. For those who had not contributed to unemployment insurance, the unemployment assistance benefit would be paid, in an amount equivalent to 120 to 150 percent of the social relief amount prescribed by the local civil affairs department, and the specific amount is determined by the provincial government.

### 3.4. Social Assistance: Abolishing the Restrictions on Household Registration and Enriching the Form of Payment

In most countries, social assistance acts as a bedrock and functions as a last resort of social protection, which aims to contribute to the prevention and alleviation of poverty. China issued the Interim Measures for Social Assistance (IMSA) in 2014, which initially established a social assistance system consisting of the minimum subsistence security system and eight specific social assistance programs. (The eight specific social assistance programs include the minimum living standard scheme, the relief and support system for people living in dire poverty, medical assistance, educational assistance, housing support, legal assistance, a relief system after natural disasters and the temporary-assistance scheme.) With the target of “precise poverty alleviation” to be achieved in 2020, the goal of the social assistance system will shift from eliminating absolute poverty to alleviating relative poverty [29].

Following the outbreak of the Covid-19 pandemic, a range of social assistance measures were taken to guarantee a basic livelihood for the poor. During the period of epidemic prevention and control, vulnerable groups, such as families receiving subsistence allowances (i.e., the *Dibao* households), low-income groups, vagrants, beggars, and workers who could not return to their workplace, encountered many difficulties as a result of regional closures, self-isolation, or quarantine. To address this situation, Chinese civil affairs departments adopted a series of measures: (1) For the *Dibao* households, the family means test could be carried out remotely, and the means test and dynamic adjustments could be suspended in areas with severe pandemics. (2) The amount of cash benefits for the impoverished increased. For instance, Hubei Province stipulated that 500 yuan ($70.6) should be added to the amount received by people in need in urban areas, and 300 yuan ($42.4) in rural areas. (3) Temporary assistance would be given to those who have difficulty in life due to self-isolation, quarantine, or infection. (4) In principle, China’s social assistance is mainly for people with local household registration (*Hukou*). However, during the Covid-19 outbreak, temporary accommodation, food, clothing, and other help was provided to non-local residents in need.

Despite the success in constraining the Covid-19 pandemic, at present, one of the major problems with social assistance in China is that each specific sub-program is based on the minimum subsistence security system. In many regions, only those households with low-income are qualified to apply for benefits from other specific assistance programs. During the Covid-19 pandemic, there were many laborers who could not return to their hometowns in time for the New Year’s holiday. Urban residents’ incomes dropped, and they experienced temporary difficulties due to their employers failing to resume work in time. These fragile groups are undergoing hardship and need temporary assistance. However, according to China’s IMSA, the objectives and standards of temporary assistance are not clear. In practice, it is mainly aimed at the homeless. But it should be also targeted at fragile groups whose lives have been severely constrained during major crisis events like the Covid-19 pandemic. Since China’s social assistance is tied to local household registration, workers and residents from other administrative areas cannot apply, which may be reasonable under normal circumstances, but in the context of major emergencies, household registration restrictions should be softened.

This scenario represents another problem in the existing social assistance system that has been exposed during the Covid-19 pandemic. Many people in need of social assistance benefits might not be living in their local household registration location, and thus cannot possibly apply in a timely manner. Thus, it is crucial to reflect on the current social assistance system in China and extend social assistance benefits to all residents in the face of sudden public health events.

Furthermore, the current social assistance system operates mainly by providing cash benefits. However, in this pandemic, most communities have adopted the strategy of closed management, and cash benefits alone might not enable the beneficiaries to purchase goods or services in time. Therefore, during the Covid-19 pandemic, social assistance must provide in-kind benefits and services and ensure the basic livelihood of Chinese residents. At the same time, special emphasis should be placed on employment assistance, as it helps people who have temporarily lost their jobs due to the pandemic become more employable as soon as possible and become self-reliant workers again.

### 3.5. Socio-Technical Adjustments and Digitalization: Performance-Enhancing Factors

In modern society, social security systems represent an important institutional arrangement in responding to individual risks through collective organization. With the proper utilization of techniques and digitalization in public services, the efficiency and effectiveness of the social security can be substantially enhanced.

First of all, a number of charitable organizations actively carry out various fundraising activities to make up for the shortage of public resources. During the pandemic prevention and control periods, charitable organizations fully utilized their advantages in terms of fundraising, social services, and mental health interventions. Various foundations mobilized social donations and purchased pandemic materials for prevention, effectively making up for the lack of materials in some significant areas in the early stages of the crisis. As of 24:00 on 8 March, charitable organizations and the Red Cross system at all levels across the country had received about 29.29 billion yuan ($4.14 billion) in donations and donated about 522 million items [24]. Among them, the Wuhan Red Cross Society (RCS) received more than 1.697 billion yuan ($0.24 billion) by 24:00 on 3 April, according to the announcement on its official website [30]. In addition, the closed management of residential areas in many regions brought great inconvenience into the daily lives of residents. Many charitable organizations have launched services such as centralized purchase of daily necessities for residents and the provision of mental health counseling to help residents survive the pandemic period.

In addition, efforts to promote and practice online activities not only improved the efficiency of the services, but also reduced the risk of pandemic spread. After the pandemic occurred, various social security agencies actively promoted online processing. Business that previously was undertaken on a face-to-face basis could be moved online, thus reducing the crowding of people, the chances of infection for various fund-contributing companies, charitable organizations, etc. Moreover, the efficiency and quality of such services were also improved. For example, the departments of Human Resources and Social Security announced an online application platform for claiming unemployment insurance benefits, notifying each recipient about their benefits online. It is believed that after the pandemic, more beneficiaries of social insurance will be accustomed to handling social security procedures or formalities online.

Nevertheless, looking back at the performance improvement of the social security system reveals some urgent and necessary adjustments. The larger and broader the impact of the public health emergency, the more actors are involved in the response, and more effective cooperation between different parties is required [31]. Among them, the most important is the cooperation between the government and charitable organizations. As the third sector, charitable organizations can provide more precise, differentiated, and high-quality services. Over the course of the pandemic prevention and control, charitable organizations not only demonstrated a strong capacity to draw resources, but also exposed problems such as insufficient credibility, untimely information disclosure, and unfair resource distribution. For example, the Wuhan RCS did not distribute donated materials to hospitals in time, there was unfairness in the process of distribution, and the information about donated and distributed materials was not disclosed in time, all of which led to a wave of questioning by the public.

Charitable organizations in China are largely still immature; therefore, to strengthen their capacity, China should focus on the following three aspects: (1) Strengthen the role of hub-type charitable organizations such as the Charity Federation, the RCS, etc., at all levels, and utilize them as a link between governments and charitable organizations to coordinate social resources in response to major public crises. (2) Acknowledge the shift in charitable organizations’ functions from financing to service provision. Charitable organizations in China are currently divided into social groups, foundations, and private non-enterprise units. Among them, foundations can both raise funds and provide social services; private non-enterprises can only provide social services and are unable to raise funds. In accordance with the principle of the social division of labor, it is recommended to establish a new pattern, with funds raised by foundations, professional services provided by social service agencies, and a connection between these two types of organizations through a bidding mechanism. (3) Information disclosure should be well implemented. In the event of a major crisis, in particular, charitable organizations should promptly release information about fundraising and material distribution through various channels and accept public supervision. Only in this way can public maintain confidence in charitable organizations.

## 4. Discussion

The unexpected and sudden outbreak of Covid-19 in Wuhan and its prompt spread nationwide have created a special situation and state of exigency and urgency, challenging the status quo of the socio-economic order within and outside Hubei province. The crisis precipitated by the pandemic has quickly led to a critical juncture at which the Chinese administration must quickly and effectively respond. The negative effects of the crisis have created strong pressure and an incentive for a quick administrative response and effective action in the part of the Chinese state. The explosiveness and severity of the pandemic crisis and its unpredictable as well as astronomical social costs have strengthened the model of “big government” in the Chinese case [32], with massive state intervention in society and the economy. Besides the usual intervention methods such as lockdowns, curfews, travel bans, the use of big data for tracing and breaking infection chains, the creation of health-related codes via smartphones, and nationwide mobilization of medical assistance for Wuhan and Hubei province, social policy intervention has also become especially relevant. In both the mid-crisis and post-crisis periods, we have observed an increase in social welfare and social protection programs. However, this topic has been largely neglected, both in academic research and public perception.

Social policy intervention is deeply connected with the social fact that modern society is a risk society [33], and some social risks, such as environmental pollution and global warming, have evolved into global risks that challenge all nation states. Social policy must circumvent new global risks like global pandemics, and the social protection programs of nation states must find an effective way to mitigate the social risks and hazards caused by newly emerging infectious diseases such as Covid-19. These programs are not only targeted at national citizens, but also at international migrants such as tourists, foreign visitors, guest workers, etc. How modern social policy responds to pandemics has become a new and urgent research question for all nation states.

The adopted social protection programs in China include a wide range of policy areas, such as health insurance, unemployment insurance, and accident insurance, among others. From the perspective of system types, we can differentiate social protection programs amid the Covid-19 crisis into social insurance, social assistance, social welfare, and enterprise-related special subsidies and policy measures. Existing social insurance programs, the MLSS, and some special temporary policy arrangements have been combined to circumvent the sharp increase in social suffering. Within the portfolio of different benefits, we have noted: (1) cash payments such as unemployment allowances and unemployment subsidies, benefits from the Chinese social assistance program (MLSS), ensuring the material security of millions of people and employees who have suffered from temporary layoffs, shortened work hours, or mandatory breaks imposed by employers; (2) benefits in kind, including service programs like testing, diagnosis, and therapy for Covid-19 patients, free of charge, either financed by health insurance programs or subsidized by state revenues. Also included are special social protection and social services for elderly people in nursing homes and social welfare units who constitute one of the highly vulnerable groups exposed to the virus; (3) favorable policy measures, such as the alleviation of income tax burdens and the granting of special loans for small- and medium-sized enterprises who face challenges in existential survival owing to a lack of liquidity because of the drastic freeze in economic and commercial activities. With regard to targeted welfare clients, we can also distinguish two types of program—individual-related and collective-related benefits. The first includes benefits that target individual demands, delivered to each citizen in case of need. The second is delivered to collective units such as households and enterprises. In sum, different interventionist forms, including monetary intervention, service-related intervention, policy-related intervention, and legal intervention have been combined and synthesized to help residents and social and economic units to traverse the “valley of tears” [34] amid the Covid-19 crisis.

## 5. Conclusions

The unprecedented Covid-19 crisis has indeed stimulated rigorous state intervention in the areas of livelihood, welfare, and wellbeing for millions of people who have suffered directly and indirectly from its negative effects. As with various historical examples, the crisis derived from the coronavirus has stimulated public expenditures and a model of “big government”. It has further legitimated hyper-normal and, in some cases, nationwide and large-scale extralegal intervention policy to lessen the intensity and severity of soaring social problems and escalating social conflicts. However, this kind of special intervention within and beyond the regular legal and institutional framework has also reached its limits, leaving many problems unresolved, such as the question of who should take responsibility for the medical treatment of residents that have dropped out from the regular health insurance system and who should finance diagnosis and therapy for overseas Chinese and foreigners returning to Chinese territory, and to what extent. The tacit problems of coverage loopholes in the Chinese social security system have become explicit during the development of the crisis. Even in cases of monetary compensation and intervention, impoverished residents or the unemployed who have received cash benefits, either from social assistance or from unemployment insurance, have been unable to purchase enough daily necessities and basic commodities due to the lockdown and curfew that has constrained the normal functioning of physical stores and brick-and-mortar businesses. Charitable organizations have played a vital role in providing benefits in kind for individuals and families in need. Ensuring material security and social safety encompasses more than one single area of social policy and requires much more coordinated policy intervention from different arenas, including economic policy, employment policy, fiscal policy, and social policy.
